# 超高效液相色谱-串联质谱法测定叔胺类药品中硫酸二甲酯基因毒性杂质

**DOI:** 10.3724/SP.J.1123.2022.01008

**Published:** 2022-09-08

**Authors:** Liping GONG, Baojian HANG, Ruiqing XIAN, Mingzheng YANG, Xunjie ZHANG, Xia WEI

**Affiliations:** 山东省食品药品检验研究院, 国家药品监督管理局仿制药研究与评价重点实验室,山东省仿制药一致性评价工程技术研究中心, 山东 济南 250101; Shandong Institute for Food and Drug Control, National Medical Products Administration (NMPA) Key Laboratory for Quality Evaluation of Gelatin Products, Shandong Research Center of Engineer and Technology for Consistency Evaluation of Generic Drugs, Jinan 250101, China

**Keywords:** 超高效液相色谱-串联质谱, 衍生, 氨基比林, 咖啡因, 替加氟, 硫酸二甲酯, ultra-high performance liquid chromatography-tandem mass spectrometry (UHPLC-MS/MS), derivative, aminopyrine, caffeine, tegafur, dimethyl sulfate

## Abstract

硫酸二甲酯被广泛用于药品的有机合成中,具有很强的毒性及腐蚀性,因此应严格控制其在药品中的残留量。现有传统检测方法在样品制备过程中不允许使用水,且存在专属性低、灵敏度差等缺陷,严重制约了检测方法的通用性和准确度。研究采用超高效液相色谱-串联质谱技术(UHPLC-MS/MS),建立了氨基比林等叔胺类药品中残留的硫酸二甲酯的测定方法。检测方法以氨基比林作为衍生剂,40 ℃条件下氨基比林和硫酸二甲酯在水溶液中能够快速反应生成甲基化氨基比林,通过检测甲基化氨基比林间接计算硫酸二甲酯的残留含量。采用Waters Atlantis HILIC C18色谱柱(100 mm×2.1 mm, 3.0 μm),以10 mmol/L乙酸铵水溶液-0.1%甲酸甲醇溶液(50∶50, v/v)为流动相进行等度洗脱,流速0.3 mL/min,柱温40 ℃,进样量1 μL;在电喷雾电离、正离子(ESI^+^)多反应监测(MRM)模式下测定硫酸二甲酯的含量。硫酸二甲酯在0.9935~7.9480 ng/mL范围内线性关系良好,相关系数(*r*)为0.9997,方法的检出限为0.50 ng/mL,定量限为1.15 ng/mL,加标回收率为94.9%~106.4%。建立的检测方法用于氨基比林、咖啡因、替加氟等9批样品中,均未检出硫酸二甲酯,说明各药品生产企业注重了该基因毒杂质残留量的控制。该方法专属性强,灵敏度高,操作简单,定量准确,适用于氨基比林等药品中硫酸二甲酯基因毒杂质的测定。

## Abstract (en)

Dimethyl sulfate is an important chemical raw material that is widely used in the synthesis of drugs, dyes, spices, and pesticides. The highly toxic and corrosive dimethyl sulfate residue in medicines is harmful to the human body, and hence, the residue level should be strictly controlled. Traditional detection methods use high-purity acetonitrile and anhydrous as the solvents, which limits the choice of detection solvents and degrades the versatility and accuracy of detection. Therefore, a simple and accurate method for the determination of dimethyl sulfate residues is urgently needed. Dimethyl sulfate is usually detected by ultra-high performance liquid chromatography-tandem mass spectrometry (UHPLC-MS/MS) with pyridine as the methylation substrate. In this study, a new method for the detection of dimethyl sulfate was established using tertiary amines such as aminophenazone, which has many advantages over pyridine, as the methylation substrate. For example, the hybrid orbital and electron cloud of the N atom are different, resulting in stronger nucleophilicity of aminophenazone. High temperatures that are detrimental to the stability of dimethyl sulfate are not required when using aminophenazone, and the aliphatic quaternary ammonium salt product is more stable, with good stability, low interference, good ionization properties, and high response. The separation was performed on a Waters Atlantis HILIC C18 column (100 mm×2.1 mm, 3.0 μm) using a mobile phase consisting of 10 mmol/L ammonium acetate solution-0.1% formic acid methanol solution (50∶50, v/v) at a flow rate of 0.3 mL/min. The column temperature was set at 40 ℃, and the sample size was 1 μL. Dimethyl sulfate was determined in the electrospray positive ionization (ESI^+^) and multiple reaction monitoring (MRM) modes. Dimethyl sulfate showed good linear relationships within the range of 0.9935 to 7.9480 ng/mL (*r*=0.9997). The limit of detection and limit of quantification for dimethyl sulfate were 0.50 ng/mL and 1.15 ng/mL, respectively. The recoveries (*n*=3)of dimethyl sulfate were 94.9% to 106.4%. The relative standard deviations (RSDs) were 1.44% to 5.51%. The RSD of the methylated aminophenazone peak area was 4.32%, indicating good stability of the reaction product. Dimethyl sulfate genotoxic impurities were not detected in 9 batches of aminophenazone, caffeine, and tegafur samples, which indicated that the drug manufacturers paid attention to the control of these impurities. The proposed method is advantageous over the existing techniques in terms of the better ion peak shape and higher molecular weight, without interference from other fragments. The method is specific, sensitive, simple, rapid, and accurate, and it can be used for the determination of dimethyl sulfate genotoxic impurities in aminophenazone and other medicines.

硫酸二甲酯是重要的化工原料之一^[1,2]^,在有机合成中作为甲基化试剂广泛用于制造二甲基亚砜、咖啡因、可待因、香草醛、安替比林、氨基比林、甲氧苄氨嘧啶等药物以及农药乙酰甲胺磷等,有些药物在生产过程中也会产生硫酸二甲酯^[3]^。硫酸二甲酯为剧毒品,具有遗传毒性和致癌性,且具有强烈的腐蚀性,能在动物体内组织中缓慢水解生成甲醇和硫酸,其毒性是由未分解的分子和水解后的生成物共同作用产生。药物中残留的硫酸二甲酯在过量(《美国药典》《欧洲药典》规定原料药中硫酸二甲酯的含量不得过0.000038%)时会对人体产生巨大的危害,因此非常有必要控制药物中硫酸二甲酯的残留量^[4,5]^。

目前对硫酸二甲酯的检测方法主要有气相色谱法、气相色谱-质谱联用法和液相色谱法^[6⇓⇓⇓⇓⇓⇓⇓⇓-15]^,但准确测定药物中残留的硫酸二甲酯含量极具挑战性,因为硫酸二甲酯极性很大,不具备敏感的紫外吸收基团,因此传统方法在专一性、灵敏度、方法精密度和重复性等方面都存在很大缺陷,且检测过程中对溶剂的要求很严格,如溶液配制不允许使用水,必须使用纯度极高的乙腈,气相色谱-质谱联用法得到的谱图在低分子端会出现大量的碎片峰,干扰检测的精准度。

本研究以氨基比林作为衍生试剂,采用超高效液相色谱-串联质谱法建立了氨基比林、咖啡因、替加氟等叔胺类药物中硫酸二甲酯的检测方法。硫酸二甲酯具有甲氧基基团,可以和氨基比林发生*N*-烃基化反应生成甲基化氨基比林(季铵盐),样品前处理简便,生成的甲基化氨基比林稳定性高、干扰小、质谱离子化好、响应高,方法快速、准确、灵敏,可用于上述叔胺类药品的质量控制。

## 1 实验部分

### 1.1 仪器与试药

AB SCIEX Triple Quad 6500+超高效液相色谱-三重四极杆质谱联用仪(AB公司;美国); XSE205电子天平(Metter Toledo公司,瑞士); KQ-300GDV型恒温数控超声器(昆山市超声仪器有限公司); Vortex-6涡旋振荡器(海门市其林贝尔仪器制造有限公司)。甲醇、丙酮(色谱纯,美国赛默飞公司),甲酸(色谱纯,美国ACS公司),乙酸铵(色谱纯,国药集团化学试剂有限公司),去离子水(18.2 MΩ·cm,由美国Merck-Millipore公司生产的Millipore Milli-Q Advantage超纯水系统制得)。对照品:硫酸二甲酯(山东西亚化学公司),纯度99.5%。样品:氨基比林、咖啡因、替加氟原料药共9批。

### 1.2 色谱、质谱条件

液相色谱条件:色谱柱为Waters Atlantis HILIC C18色谱柱(100 mm×2.1 mm, 3.0 μm),以10 mmol/L乙酸铵水溶液-0.1%甲酸甲醇溶液(50∶50, v/v)为流动相进行等度洗脱,流速0.3 mL/min,柱温40 ℃,进样量1 μL。

质谱条件:电喷雾电离(ESI)源,正离子扫描模式,多反应监测;离子源温度:550 ℃。定性、定量离子对、锥孔电压、碰撞能量见表1。

**表 1:** 甲基化氨基比林的质谱采集离子信息

Compound	Parent ion (m/z)	Product ion (m/z)	Cone voltage/V	Collision energy/eV
Methylated	246.2	97.1	40	35
aminopyrine		230.2^*^	40	45

* Quantitative ion.

### 1.3 衍生剂、空白溶液的制备

取氨基比林1.000 g,精密称定,置于100 mL量瓶中,加水溶解并稀释至刻度,摇匀,作为衍生剂溶液;取0.5 mL水,加衍生剂溶液0.5 mL, 40 ℃反应2 h,作为空白溶液。

### 1.4 对照品溶液的制备

取硫酸二甲酯对照品适量,精密称定,加丙酮溶解并定量稀释,制成20 μg/mL的对照品储备溶液。精密量取溶液0.05 mL,加水稀释制成4 ng/mL的对照品工作溶液(临用新制);精密量取溶液0.5 mL,精密加入衍生剂溶液0.5 mL,摇匀,40 ℃反应2 h,作为对照品溶液。

### 1.5 供试品溶液的制备

氨基比林原料药:取本品适量,精密称定,加水溶解并定量稀释,制成5 mg/mL的溶液;取溶液1.0 mL,于40 ℃放置2 h,作为氨基比林供试品溶液。

替加氟等叔胺类原料药:取本品适量,精密称定,加水溶解并定量稀释,制成5 mg/mL的溶液;精密量取溶液0.5 mL,精密加入衍生剂溶液0.5 mL,摇匀,40 ℃反应2 h,作为其他叔胺类供试品溶液。

## 2 结果与讨论

### 2.1 反应产物的确认及反应温度的考察

硫酸二甲酯是甲基化试剂,和氨基比林发生*N*-烃基化反应生成季铵盐(见图1)。反应过程中温度是影响反应效率的主要因素,因此对不同反应温度进行了考察。

**图 1:**
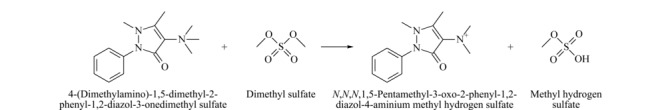
硫酸二甲酯与氨基比林的反应式

考察实验过程如下:精密称定硫酸二甲酯(相对分子质量为126.13)适量,加丙酮溶解并稀释制成浓度为4.5 mmol/L的溶液。精密称定氨基比林(相对分子质量为231.29)适量,加水溶解并稀释制成浓度为45.0 mmol/L的溶液。取上述硫酸二甲酯溶液和氨基比林溶液各1 mL,混匀,分别于30、40、50 ℃反应2 h,取上述反应液各1.0 mL,分别置于100 mL量瓶中,用水稀释至刻度,摇匀,作为考察样品溶液。同法制备空白溶液。采用Thermo QE Plus高分辨质谱联用仪进行分析,计算出不同温度下的反应效率,并对反应产物进行定性确认。

考察样品溶液和空白溶液的一级高分辨离子色谱图和紫外光谱图分别见图2a和图2b。考察样品溶液中因氨基比林在反应体系中过量,故有剩余没反应的氨基比林,因此会采集到衍生产物甲基化氨基比林和剩余反应物氨基比林的质谱信息,同时采集了剩余反应物氨基比林的紫外光谱;空白溶液中只有氨基比林,因此一级高分辨离子色谱图只有氨基比林的信息。由于采用的是高分辨质谱,精度高,可以对反应产物进行定性确认,由一级高分辨离子色谱图可以看出,氨基比林与硫酸二甲酯的反应液中检测到反应产物的精确质量数为246.1246,与氨基比林甲基化产物的理论精确质量数246.1246一致,确认了反应产物为甲基化氨基比林。以氨基比林在考察样品溶液和空白溶液中233 nm测定的紫外光谱图峰面积差值计算反应效率。随着温度的升高,硫酸二甲酯和氨基比林的反应效率增加,在40 ℃时基本上达到完全反应,因此选择40 ℃作为衍生温度。

**图 2:**
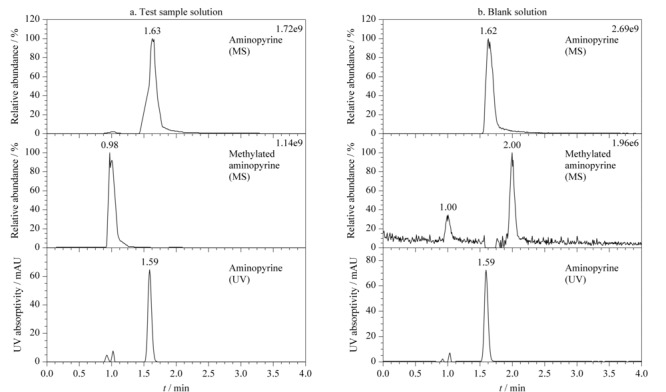
反应物、反应产物在(a)考察样品溶液和(b)空白溶液中的提取离子色谱图和紫外色谱图

### 2.2 氨基比林衍生剂的选择

吡啶是硫酸二甲酯发生甲基化反应的常用底物,是一个含有氮杂原子的六元杂环化合物,具有芳香性,而本研究发现的氨基比林为含有*α*,*β*不饱和酮结构的脂肪胺,吡啶环上的*N*原子相比氨基比林上的*N*原子因电子云轨道杂化、空间位置不同而活性较低,且硫酸二甲酯与胺的反应属于双分子亲核取代(SN2)反应,在SN2反应中,氨基比林表现出更强的亲核性,反应能够快速进行。与吡啶相比,氨基比林与硫酸二甲酯反应不需要高温,较低温度有利于硫酸二甲酯的稳定。吡啶与硫酸二甲酯反应后产物具有季铵盐的特性,但生成的季铵盐不利于其芳香性的保持,不够稳定,但是氨基比林生成的脂肪胺季铵盐稳定性好,因此本研究选取的氨基比林更适合作为甲基化试剂用于硫酸二甲酯的检测。

### 2.3 专属性

精密量取空白溶液、对照品溶液、供试品溶液各1 μL,分别进样测定。空白溶液与供试品溶液中其他杂质峰均不干扰硫酸二甲酯衍生后的甲基化氨基比林的检测,空白溶液中甲基化氨基比林保留时间处有峰值很小的本底色谱峰(见图2)。连续将空白溶液进样7次,计算该色谱峰峰面积的RSD值为6.17%,表明该色谱峰的稳定性良好,不影响硫酸二甲酯的检测。

### 2.4 标准曲线

分别精密量取对照品储备溶液(20 μg/mL)0.5、1.0、2.0、3.0、4.0 mL,分别置于100 mL量瓶中,加水稀释至刻度,摇匀。分别精密量取上述溶液0.5 mL,再分别精密加入0.5 mL衍生剂溶液,混匀,40 ℃反应2 h,进样测定。以甲基化氨基比林的峰面积为纵坐标(*y*),各溶液的质量浓度为横坐标(*x*, ng/mL),绘制标准曲线,结果表明在0.9935~7.9840 ng/mL范围内,线性关系良好,回归方程为*y*=17901*x*-3146,相关系数(*r*)为0.9997。

### 2.5 检出限和定量限

基于空白溶液中存在本底峰,检出限和定量限采用《中国药典》2020年版四部通则9101中“基于响应值标准偏差和标准曲线斜率法”测定。取空白溶液连续进样7次,计算检出限为0.50 ng/mL(0.05 ppm),定量限为1.15 ng/mL(0.115 ppm)。

### 2.6 精密度

取对照品溶液连续进样6次,甲基化氨基比林峰面积的RSD值为2.80%,表明该方法的仪器精密度良好。

### 2.7 稳定性

取对照品反应溶液分别在0、2、4、6、20 h进样测定,甲基化氨基比林峰面积的RSD值为4.32%,表明反应产物稳定性良好。

### 2.8 回收率

本方法通过向阴性氨基比林、替加氟样品中分别添加低、中、高3个水平的硫酸二甲酯对照品溶液,每个水平进行3次平行试验,计算方法回收率,结果见表2。硫酸二甲酯的平均回收率为94.9%~106.4%,相对标准偏差为1.44%~5.51%,满足实际样品的检测需求。

**表 2:** 三个水平下硫酸二甲酯的加标回收率(*n*=3)

Sample	Spiked/ (μg/kg)	Detected/ (μg/kg)	Recovery/ %	RSD/ %
Aminopyrine	1.582	1.684	106.4	1.92
	1.977	1.877	94.9	1.44
	2.372	2.281	96.2	2.47
Tegafur	1.582	1.602	101.3	5.51
	1.977	1.999	101.1	3.87
	2.372	2.324	97.9	4.10

### 2.9 样品测定

取氨基比林、咖啡因、替加氟共9批原料药按1.5节制备供试品溶液,进样测定。按外标法计算硫酸二甲酯的含量,9批样品中均未检出该杂质,表明氨基比林、咖啡因、替加氟原料药生产企业在生产工艺对硫酸二甲酯基因毒杂质控制良好。

## 3 结论

本文建立了超高效液相色谱-串联质谱测定氨基比林等叔胺类药品中硫酸二甲酯基因毒杂质含量的检测方法,该方法将氨基比林作为衍生剂,与药品中残留的硫酸二甲酯发生反应生成甲基化氨基比林,通过检测甲基化氨基比林间接测得药品中硫酸二甲酯的含量,进行了方法学验证,并将方法用于实际样品的测定。与现有技术相比,该方法离子峰峰形好,无其他碎片杂峰干扰,方法灵敏度高、精密度和重复性好,结果可靠,可用于氨基比林等药品中硫酸二甲酯基因毒杂质的测定。本研究有助于药品生产企业对生产工艺中基因毒杂质的控制,同时为药品监管提供技术支撑。
